# Active biosynthesis of gold nanoparticles mediated by obligate methylotrophic bacteria *Methylophilus* sp.

**DOI:** 10.3389/fmicb.2026.1852773

**Published:** 2026-06-12

**Authors:** Daniil Poberezhniy, Alexandra Kuzmitskaya, Alexander Sachavskii, Sergei Kalenov, Alexey Belov, Marina Sukhacheva, Tatyana Kolganova, Alexander Morozov, Pavel Ivanov, Mariia Gordienko, Elizaveta Mokhova, Daria Zavoiko, Viktoriia Gosteeva, Vladimir Sorokin, Dmitry Skladnev

**Affiliations:** 1 Department of Biotechnology, Mendeleev University of Chemical Technology of Russia, Moscow, Russia; 2 Research Center of Biotechnology, Institute of Bioengineering, Russian Academy of Sciences, Moscow, Russia; 3 Department of Technology of Inorganic Substances and Electrochemical Processes, Mendeleev University of Chemical Technology of Russia, Moscow, Russia; 4 Mendeleev Center for the Collective Use of Scientific Equipment, Mendeleev University of Chemical Technology of Russia, Moscow, Russia; 5 Department of Chemical and Pharmaceutical Engineering, Mendeleev University of Chemical Technology of Russia, Moscow, Russia; 6 Winogradsky Institute of Microbiology, Research Center of Biotechnology of the Russian Academy of Sciences, Moscow, Russia

**Keywords:** antimicrobial activity, gold nanoparticles, green synthesis, *Methylophilus* sp., methylotrophic bacteria, microbial synthesis

## Abstract

Obligate methylotrophic bacteria are traditional biotechnological producers due to their ability to grow rapidly in extremely simple growth media. The aim of this study was to isolate a highly efficient methylotrophic producer from natural sources specifically for the “green” synthesis of metal nanoparticles. It was demonstrated that a pure bacterial culture of the genus *Methylophilus* is the most technologically advanced for the active biosynthesis of gold nanoparticles (AuNPs). The optimal conditions for AuNPs biosynthesis were pH 5.5, 25–28 °C, 0.45 mM HAuCl_4_ and cells in the active growth phase. Under these conditions, bacteria of the genus *Methylophilus* synthesized small extracellular gold nanoparticles with a gold core measuring 4–14 nm in 6 h with a yield of up to 15 ± 3%. Continued cultivation under the same conditions for 15 h resulted in a maximum nanoparticle yield of up to 37 ± 6% with a gold core measuring 9–24 nm. After 15 h of active synthesis phase, the formation of reduced gold occurred intracellularly. Extracellular AuNPs exhibited high antioxidant activity and were characterized using FT-IR spectroscopy, SEM, TEM, XRD, and DLS. The nanoparticles shell, at least twice the size of the core, consists primarily of proteins such as porins and flagellin. The increase in the concentration of AuNPs covered with a characteristic shell outside the cells, but in the active phase of their growth, indicates a specific mechanism of their synthesis.

## 1 Introduction

Progress in nanotechnology requires an advanced set of nanotools with defined and specific properties. Nanoparticles, as fundamental building blocks, are intensively studied yet remain incompletely understood. There are problems in understanding the intricacies of their synthesis mechanisms, the complexity of the nanostructure, as well as internal and external interactions that are difficult to take into account (Ghosh et al., 2021; Singh et al., 2024; Campaña et al., 2023; Slavin et al., 2017; Nies, 2016; Bütof et al., 2018).

Among various nanoparticles, gold nanoparticles (AuNPs) hold a special position. Their unique physicochemical and optical properties, along with high biocompatibility, enable applications in medicine, electronics, and environmental science (Bedford et al., 2012; Sarfraz and Khan, 2021; Menon et al., 2017; Lin et al., 2025). In the biomedical field, the bivalence of gold atoms increases the potential for AuNPs to be used for targeted drug delivery and as contrast agents in imaging techniques (Dheyab et al., 2022). AuNPs have been shown show antimicrobial activity against resistant bacteria and pathogenic fungi (Zhang et al., 2015; Mandhata et al., 2023; Arafa et al., 2018; Gu et al., 2020). The natural optical properties of nanoparticles (in particular, surface plasmon resonance) enable the use of AuNPs is exploited for pollutant removal in water remediation (Zhu et al., 2014; Singh et al., 2018). These facts drive interest in sustainable, biogenic synthesis of AuNPs using microorganisms like bacteria and fungi (Hammami et al., 2021; Bhatnagar and Aoyagi, 2025).

Traditional chemical and physical methods for synthesizing nanoparticles often use toxic compounds and are energy-intensive, raising environmental and scalability concerns (Mikhailova, 2021). In contrast, biosynthesis in the presence of microorganisms leverages natural cellular detoxification pathways, aligning with “green” chemistry principles (Gadd, 2010). Such nanobiotechnological methods for producing nanoparticles can be easily implemented in accordance with environmentally friendly production requirements with minimal environmental impact, earning them the nickname “green” synthesis. A key task is finding or engineering fast-growing, highly productive microorganisms for efficient nanoparticle production under mild conditions in inexpensive media (Hulkoti and Taranath, 2014).

Bacteria exhibit remarkable capabilities for metal nanoparticle biosynthesis. Their metabolic pathways reduce metal ions to elemental forms, often stabilizing nanoparticles with functional biomolecules like proteins and polysaccharides (Ali et al., 2019; Narayanan and Sakthivel, 2010; Kang et al., 2017). This versatility allows control over nanoparticle size and morphology (Tukova et al., 2023; Concórdio-Reis et al., 2023).

The synthesis of nanoparticles by microorganisms can occur through intracellular or extracellular mechanisms. Intracellular mechanisms of biosynthesis may include the binding of gold ions to components of the cell wall and/or capsule, the processes of ion import into the periplasm or cytoplasm through the membrane, partial or complete reduction of gold ions, the transport of ions with an intermediate oxidation state from the cytoplasm to the periplasm in Gram-negative bacteria and their reduction there (Roy et al., 2022; Setia et al., 2025). Extracellular synthesis can occur with the participation of substances with reducing properties as well as of enzymes secreted by microorganisms, which can reduce Au^+3^ to Au^0^ (Menon et al., 2017; El-Bendary et al., 2022). In the context of extracellular synthesis, a reasonable hypothesis is the potential involvement of bacterial outer membrane vesicles (OMVs) in nanoparticle biogenesis. It can be speculated that Gram-negative bacteria, as part of a stress response, may use these vesicles to sequester and export biogenic nanoparticles, thereby protecting the cell from toxic ions (Schwechheimer and Kuehn, 2015; Mozaheb and Mingeot-Leclercq, 2020).

However, the majority of studies in this field are focused on the use of cell-free systems lacking growing microorganisms (Della-Flora and de Andrade, 2023; Carrapiço et al., 2023; Ullah et al., 2023). Moreover, biosynthesis is often considered under specifically created conditions (e.g., elevated temperatures) that are distant from physiological (Della-Flora and de Andrade, 2023; Lengke et al., 2011; Krishnan et al., 2016; El-Naggar et al., 2023; Camas et al., 2018). Consequently, the natural cellular mechanisms involved in gold nanoparticle formation, which require the presence of an active producer in the environment, may be overlooked.

The nuances of the processes and proposed mechanisms of AuNPs biosynthesis under physiological conditions for cells are insufficiently described in the literature, although substantial studies have been undertaken (Reith et al., 2009; Menon et al., 2017; Ansari and Rehman, 2021; Al-Khattaf, 2021; Gahlawat and Choudhury, 2019). To further advance research, systems with a minimum of interfering and interacting factors are needed, where the biological aspects of synthesis can be specifically identified.

The physicochemical characteristics of biogenic AuNPs depend on cultivation conditions and medium composition. While temperature and pH are easily controlled, medium composition critically affects size and uniformity (Chopra et al., 2022).

Methylotrophic microorganisms, particularly those of the genus *Methylophilus*, which can grow on synthetic media with minimal mineral components, are of particular interest for studies related to the mechanisms of synthesis and as potential producers of metal nanoparticles on an industrial scale (Sorokin et al., 2019). Members of this genus are obligate or facultative methylotrophs with a streamlined metabolism centered on the ribulose monophosphate (RuMP) cycle (Chistoserdova and Kalyuzhnaya, 2018). Their physiological resilience is supported by robust stress response mechanisms. Most methylotrophic cultures are characterized by high tolerance to heavy metals and physiological resistance to oxidative stress also, which is necessary for survival in conditions where metal nanoparticles are generated. The rapid growth and relatively simple metabolism of *Methylophilus* make methylotrophic cultures of this genus excellent candidates for studying the fundamental mechanisms of gold reduction to address these challenges.

Biotechnological synthesis involves cation reduction in the presence of growing cells, where all medium components and cellular secretions interact with ions. Developing a process requires optimizing both producer culture conditions and the chemical reduction environment. Crucially, the initial stages of low-atom cluster formation determine final nanoparticle crystalline parameters. The producer’s physiological state, influencing secretion levels and composition, significantly impacts the resulting nanoparticles’ characteristics.

Thus, nutrient medium composition and cultivation parameters essentially determine the size and homogeneity of biogenic nanoparticles. As most bioprocess parameters (temperature, pH, ion concentration, medium composition) are controllable, it becomes possible to maintain optimal conditions for reduction and precisely tune nanoparticle properties *in situ*.

Most industrial microbial producers use complex, undefined rich media. In contrast, methylotrophic bacteria grow on defined minimal media with a C_1_ source, where all components and their ratios can be precisely regulated. Therefore, in such systems, the primary reducing agents for cations are compounds secreted by the cells. This controllability, combined with the low cost and availability of methanol, positions methylotrophic bacteria as promising producers of metal nanoparticles with industrial potential and as excellent model systems for studying microbial gold reduction mechanisms (Hou, 2018).

In this study, we investigated for the first time the formation of gold nanoparticles by pure culture methylotrophic bacteria (specially isolated from natural samples) under physiological conditions. The aim of this work was to identify the relationship between cultivation parameters, the physiological state of the cells, and the physicochemical properties of the synthesized gold nanoparticles. During the study, we optimized conditions for the most efficient generation of AuNPs of the required size and hypothesized the role of outer membrane vesicles (OMVs) in the mechanism of their extracellular synthesis and stabilization as a specific response of the producer cells to gold ion-induced stress.

## 2 Materials and methods

### 2.1 Isolation of methylotrophs from natural sources and identification of bacterial cultures

Samples for subsequent isolation of methylotrophic bacteria were collected from the root zone of endemic pine growing on the seashore, which is frequently flooded and exposed to temperature and salinity fluctuations, from a depth of 15 cm (coordinates: 43.154681, 40.331367), from mucus-covered boulders of a grotto located in a mountainous area (coordinates: 43.478496, 40.548453) and from suspended silt in the marshy area of Losiny Ostrov Park (coordinates: 55.905678, 37.773028). For the cultivation of microorganisms, a modification of the “P” medium (Galchenko, 2001) of the following composition was used, g/L: KNO_3_ – 1; MgSO_4_ × 7H_2_O – 0.2; CaCl_2_ – 0.02; Na_2_HPO_4_ – 0.46; KH_2_PO_4_ – 0.35; Na_2_EDTA – 0.005; FeSO_4_ × 7H_2_O – 0.002; CoCl_2_ × 6H_2_O – 0.0002; CuSO_4_ × 5H_2_O – 0.0001; ZnSO_4_ × 7H_2_O – 0.0001; Na_2_MoO_4_ × 2H_2_O – 0.00003; MnCl_2_ × 4H_2_O – 0.00003; NiCl_2_ × 6H_2_O – 0.00002. Concentrated solutions of phosphates and microelements were sterilized separately, after which all components were mixed in one container and the only carbon source, methanol at a concentration of 6 mL/L, was sterilely added.

Enrichment and individual bacterial cultures were cultivated using “P” medium in 100- and 250-mL flasks, 1/3 full, in a Minitron Infors-HT thermostatically controlled shaker (Switzerland) at 150 rpm and 25–30 °C for up to 3 days.

Continuous cultivation in the chemostat regime was carried out in a Fermus-3 fermenter with a total volume of 5 L and a working volume of 2 L (Research Center “Bioavtomatika,” Nizhny Novgorod, Russia) with intensive aeration (one volume of air per volume of medium per minute) with an increasing dilution rate for the selection of fast-growing bacterial cultures.

The isolation of individual microorganisms from the obtained microbial communities was carried out using the Koch and depletion streak methods.

Identification of microorganisms was carried out using 16S rRNA analysis. DNA was isolated by the modified method of alkaline DNA extraction by Birnboim and Doly (1979) and the Wizard technology (Promega, United States). Polymerase chain reaction (PCR) and subsequent sequencing of the fragments of the 16S rRNA gene were performed with a universal primer system (Lane, 1991). Amplification products were sequenced by the Sanger method (Sanger et al., 1977) using a Big Dye Terminator v. 3.1 reagent kit (Applied Biosystems, Inc., United States) in an ABI PRIZM 3730 genetic analyzer (Applied Biosystems, Inc., United States) according to the manufacturer’s instructions. Initial comparison of the newly obtained sequences with the sequences from the GenBank database was performed using the NCBI Blast software.^1^ The sequences were edited using BioEdit program. The fragments of the 16S rRNA gene sequences of more than 1,400 bp were obtained for all of the isolated strains. Phylogenetic relationship dendrograms were plotted using the nearest neighbor method and the TreeViewer program (v 2.2.0). The nucleotide sequences of the microorganisms used in this study have been deposited in the GenBank database under accession numbers PZ476803, PZ476804, and PZ476805.

### 2.2 Cultivation of microorganisms and obtaining of gold nanoparticles

Pure cultures of microorganisms were cultivated on an ELMI S-3.10 M orbital shaker (Latvia) at 150 rpm and 25–30 °C for up to 3 days using a modified “P” medium in 25- and 100-mL flasks with different fillings of nutrient medium to test the effect of aeration. After cultivation, the pH was quickly adjusted to the desired value with NaOH and HCl solutions, and HAuCl_4_ was added at concentrations ranging from 0.25 to 1 mM. Observation of the synthesis, which occurred under diffused light and at temperatures of 4, 10, 20, 25–30, 37, 50 °C, continued for a week.

The analysis of the viability of cells of the studied culture producing nanoparticles after the introduction of a gold source was carried out by standard plating of cell suspensions on agar media (Koch micromethod) with methanol as the sole source of carbon and energy.

To isolate extracellular AuNPs, samples were centrifuged at 7,000 rpm for 10 min. Subsequent purification from medium components was performed using an ultracentrifuge at 25,000 rpm for 2 h. The pellet of AuNPs was resuspended in distilled water and centrifuged again. This last step was repeated at least three times.

### 2.3 Optimization of growth phase, pH, temperature and HAuCl_4_ concentration

Experiments were conducted in 12-well plates using an ELMI S-3.10 M orbital shaker (Latvia) at 350 rpm. Wells were filled to 1–2 mL. Mass cultivation of various microorganisms at different temperatures was conducted in the plates themselves as part of the optimization process. Preliminary studies to determine the optimum temperature for culturing the microorganism cultures themselves were conducted at 4, 10, 20, 25–30, 37, and 50 °C for 3 days. Subsequently, the temperature range could be narrowed to the optimum, which was determined by the growth rate and biomass accumulation, and the microorganisms were further cultivated, mainly in flasks for producing large-volume suspensions.

The resulting microbial suspension in flasks at different growth phases was collected at 2-h intervals, and aliquots were used in a series of AuNP synthesis experiments. In these experiments, the pH of the collected samples was varied using NaOH and HCl solutions in the range from 3 to 11, and the HAuCl4 concentration was varied in the range from 0.25 to 1 mM, and the suspensions were transferred to microplate wells. Synthesis was carried out at 4, 10, 20, 25–30, and 50 °C. To isolate extracellular AuNPs, samples were centrifuged at 7,000 rpm for 10 min. AuNP content was determined spectrophotometrically. Synthesis was monitored for a week.

### 2.4 Characteristics of AuNPs

#### 2.4.1 Spectral analysis in the UV–visible range

The change in the color of the samples to shades of purple upon the addition of HAuCl_4_ indicated the onset of AuNPs synthesis. To compare the obtained AuNPs samples, the primary analysis was performed using a Shimadzu UV-2600 spectrophotometer (Japan) in the wavelength range from 190 to 900 nm. The specific peak for the plasmon resonance of AuNPs and the position of its maximum in the range of 500–540 nm depends on the size of the core and can shift during the biosynthesis of nanoparticles.

#### 2.4.2 Fourier transform infra-red spectroscopy

To obtain IR spectra, pre-dried AuNPs samples were thoroughly mixed with KBr powder (a mixture of 1% sample and 99% KBr) and pressed into tablets. IR spectra were measured using a Nicolet 380 Fourier transform infra-red spectroscopy (FT-IR) spectrometer (Thermo Fisher Scientific Inc., Waltham, MA, United States) in the range of 500–4,000 cm^−1^ with a resolution of 1 cm^−1^.

#### 2.4.3 Electron microscopy

The fine structure of the samples was examined by transmission electron microscopy (TEM) using a JEM-1400 (JEOL, Japan) equipped with an energy-dispersive (EXD) X-ray spectrometer (Oxford Instruments, UK). Bright-field images were acquired at an accelerating voltage of 80 kV. Aliquots of 5 μL of each sample were deposited onto standard copper grids (grid size 200 × 200 μm) and air-dried for 15 h at room temperature. TEM images were analyzed with LabVIEW IMAQ Vision (National Instruments, United States) to build particle-size distribution histograms; at least 1,000 nanoparticles were measured for each analysis. The morphology of AuNPs was studied by scanning electron microscopy (SEM) using a JSM-6510LV (JEOL, Japan) at the D. I. Mendeleev center for collective use. Samples (5 μL) were placed on a brass stub, air-dried at room temperature, and sputter-coated with an 8 nm platinum layer in a JFC-1600 magnetron sputter coater (JEOL, Japan). SEM micrographs were acquired in secondary electron (SE) mode at an accelerating voltage of 15 kV.

#### 2.4.4 X-ray diffraction

The crystalline structure of the samples was examined by X-ray diffraction (XRD) on a DX-2700 diffractometer (Haoyuan, China) equipped with a Mythen 2R 1D position-sensitive detector (Dectris, Switzerland) and a rotating sample stage to minimize texture effects. XRD patterns were processed using JADE 9 (MDI, United States) with the ICDD PDF-4 + electronic database. The coherent scattering region (CSR) size was estimated using the Scherrer equation: d = (K∙λ)/(β∙cos*θ*), where K is the dimensionless shape factor (0.9); λ is the X-ray wavelength (1.5406 Å); β is the integral peak breadth; and θ is the diffraction angle.

#### 2.4.5 SDS-PAGE electrophoresis

Sodium dodecyl sulfate-polyacrylamide gel electrophoresis (SDS-PAGE) was implemented according to the method of Laemmli (1970) in a 12% polyacrylamide gel with using a Mini-Protean Tetra cell (Bio-Rad Laboratories, Inc.) for analysis of the molecular weight of the protein molecules, adsorbed on the surface of the nanoparticles. Proteins were washed off from the nanoparticles by boiling them in a 2% SDS solution for 20 min. Then the protein sample (20 μL) was mixed with an equal volume of twofold Sample Buffer, refluxed in a water bath for 10 min, loaded into the gel well in a volume of 20 μL, and electrophoresis was performed at a constant voltage of 170 V. The mixture of protein markers PageRuler Prestained Protein Ladder 10–180 kDa (Thermo Scientific, United States) was used as a molecular mass standard. The gel was stained with silver nitrate/Coomassie Brilliant Blue and visualized for documentation using the GelDoc Go Imaging System (Bio-Rad Laboratories, Inc.).

#### 2.4.6 Identification of proteins that make up the AuNPs shell

The composition of the AuNPs shell proteins was determined using MALDI mass spectrometry as described in Shaposhnikov et al. (2023).

Identification of the proteins forming the AuNPs shell was performed by correlating the obtained tandem mass spectra with databases constructed from the amino acid sequences corresponding to protein-coding genes identified *ab inito* in the *Methylophilus* sp. F5P3.1 m genome. To identify the AuNPs shell proteins produced using *Methylophilus* sp. F5P3.1 m, a set of translated coding sequences obtained by annotating the whole genome sequence of this microorganism was used.

#### 2.4.7 Antimicrobial properties of AuNPs: minimal inhibitory concentration/minimum bactericidal concentrations

The cultures used in this work were the following: *Pseudomonas aeruginosa* PA01 4/4–15, *Staphylococcus aureus* 209p, *Bacillus cereus* B 504T UNIQEM, *Escherichia coli* K-12.

To quantitatively evaluate the antibacterial activity of biogenic metal NPs, the minimum inhibitory and biocidal concentrations (MIC and MBC, respectively) of the particles were determined using the dilution antimicrobial susceptibility test (Cockerill et al., 2012). To conduct the study, overnight cultures of model pathogenic microorganisms grown in LB medium at 37 °C were used. Suspensions of the test microorganisms were standardized by adjusting their optical density to values corresponding to the McFarland turbidity standard of 0.5 (equivalent to ~1 × 10^8^ cells/mL) by diluting with fresh sterile growth medium. The resulting standardized bacterial suspensions were used to inoculate 96-well culture plates, each well of which contained 150 μL of LB medium with the addition of one of the tested biocidal agents: AgNO_3_ (positive control) or AuNPs. The following concentrations of antimicrobial agents were chosen for testing: 5, 10, 25, 50 and 100 (mg Ag in AgNO_3_)/L and 5, 10, 25, 50, 100, 150, 200, 250 and 300 (mg Au^0^)/L for AuNPs. Serial dilutions of the test agents were carried out by successive dilution of their concentrates with LB medium. Native LB medium without the addition of any biocidal agents served as a negative control. The selected concentrations of each antimicrobial agent corresponded to three wells of the culture plate. Inoculated plates were cultured for 24 h on an orbital shaker at 350 rpm and 37 °C in an air thermostat. After completion of cultivation, MIC determination was carried out by visual assessment of the growth of pathogen cultures. The MIC was considered to be the minimum concentration of a biocidal agent at which no visible growth of microorganisms was observed. Next, the number of colonies forming units (CFU) was determined from the wells of the plate in which inhibition of culture growth occurred. CFU was determined using the plate method (Koch method) by seeding on LB agar medium. The minimum concentration of the biocidal agent in a well from which no colonies of the test culture were formed was taken as the MBC. In cases where the MIC or MBC differed for different experimental replicates, the highest of the obtained concentrations was taken as the true one.

#### 2.4.8 Antioxidant activity of AuNPs

To determine the antiradical activity (AOA) of our preparations, the reaction with a stable free radical 2,2-diphenyl-1-picrylhydrazyl (DPPH) was used (Molyneux, 2004). The studies were carried out using a Shimadzu UV-2600 recording spectrophotometer at a wavelength of 517 nm in 1 cm wide cuvettes. A solution of DPPHs (0.2 mmol/L) manufactured by Aldrich was prepared in 96% ethanol. To 2 mL of each of the obtained series solutions, 2 mL of the DPPH solution was poured and mixed after 30 min or another specified time; the optical density values were recorded at λ = 517 nm. As control samples, pure solvents were used to prepare working solutions. Working solutions were prepared using sequential dilutions. Only freshly prepared solutions were used in this study. All solutions that developed turbidity were centrifuged at 20 °C for 20 min at 14,000 rpm. The formula was calculated as % inhibition. Inhibition % = (A _C(0)_–A _A(t)_/A _C(0)_)·100, where A _C(0)_ is the optical density of the control sample, A_A(t)_ – optical density of the sample under study. One antioxidant activity unit is the amount of a substance (IC_50_ – concentration of a substance) that causes 50% inhibition of a DPPH solution under special conditions. The IC_50_ value was calculated from the linear region of the inhibition percentage versus antioxidant amount curve, accounting for the solution color.

#### 2.4.9 ICP-OES analysis and nanoparticle yield

To determine the concentration of gold in nanoparticles, a sample was quantitatively introduced into a polypropylene test tube, then 3 mL of aqua regia was added to the test tube and the contents of the test tube were mixed until the sample was completely dissolved. Next, the contents of the test tube were brought to a volume of 20 mL with freshly prepared deionized water (18.2 MOhm∙cm) and thoroughly mixed. The resulting solutions were analyzed on an Agilent 5800 VDV ICP-OES optical emission spectrometer (Agilent Technologies, Malaysia). The plotting of calibration dependencies was carried out using standard samples prepared by successive dilution based on the multi-element standard MES-3 (“SKAT” Scientific and Production Enterprise LLC, Russia). The acids used during sample preparation were pre-purified by sub-distillation using the SubPUR acid purification system (Milestone, Switzerland). The yield of AuNPs was calculated from the difference between the amount of HAuCl_4_ introduced and the purified AuNPs obtained.

#### 2.4.10 Statistical analysis and visualization

All experiments were performed in triplicate, and data are presented as mean ± standard deviation. Visualization of the obtained data was performed using R package shiny (v1.10.0) and GraphPad Prism (v8.0.1).

## 3 Results

### 3.1 Selection of methylotrophic microorganisms capable of reducing au^+3^ under cultivation conditions

The synthesis of gold nanoparticles using microorganisms and other biological systems depends on many factors, such as pH, temperature, and the amount of gold precursor added. But the most important factor influencing the ability of living producers to synthesize nanoparticles is the physiological state of their cells, which, in turn, depends on the conditions of previous cultivation. When developing a biotechnological process, conditions are optimized that ensure the optimal regime of both the cultivation process itself and the optimal composition of the medium with a certain ratio of components capable of influencing the enzymatic activities of the producer. Often, some of the listed parameters change during the cultivation process, may depend on the stage of cell growth and thus affect the level of synthesis of various enzymes, metabolites, and secreted compounds (Tikariha et al., 2017; Cardoso et al., 2025; Mohanta et al., 2020; Santhosh et al., 2022). When selecting microorganisms for green production of metal nanoparticles, it is necessary that the cells be capable of generating reducing equivalents for gold reduction at a certain rate. At the same time, to stabilize the nanoparticles, the reaction mixture must contain certain proteins, polysaccharides and other compounds capable of protecting the metal core from enlargement (Cardoso et al., 2025; Kazemi et al., 2023; Lee et al., 2020). The listed parallel processes developing during the biosynthesis of nanoparticles limit the possibilities of choosing effective microbial producer cultures.

This work began with the search for efficient C_1_-utilizing microbial producer cultures specifically for the production of gold nanoparticles. Our experience has shown that among several hundred methylotrophic bacteria isolated from various natural sources, only a few are found that can reduce gold under certain conditions. Only a few cultures are capable of performing this reduction rapidly and with a significant yield of long-lasting, stable nanoparticles. Among the bacteria selected, those whose cells were capable of extracellular biosynthesis of gold nanoparticles were of greatest interest.

Isolation of actively growing methylotrophs was carried out from samples taken in specific ecological niches, the conditions of which facilitate the survival of methylotrophic microorganisms that are resistant to natural stress and are part of methane-dependent or plant-associated communities (Taubert et al., 2019; Gogleva et al., 2010; Yang et al., 2020). Samples collected at the exits of grottoes and caves, from bogs with variable light and temperature conditions, and from biofilms/fouling on the surface of rocks over which streams, sometimes containing gasses, flowed were used for inoculation on C_1_ growth media. The root zone of endemic pine trees growing on the frequently flooded seashore was also a source of samples.

In the first stage, a standard method for obtaining enrichment cultures in flasks was used. To select fast-growing monocultures, a continuous cultivation method with increasing dilution rate and subsequent plating on solid media was employed.

The possibility of synthesizing gold nanoparticles by the isolated bacteria was then tested in flasks at different pH, in the presence of different concentrations of HAuCl_4_ as a source of gold cations, at different stages of culture growth, and at the optimal cultivation temperature. The selection of promising isolates was carried out on the basis of staining of cell suspensions directly in the growth medium. The level of biosynthesis of gold nanoparticles and the selection of promising isolates was based on the change in the color of the cell suspension, which could change from bright red to lilac (λ 520–550 nm), then to dark lilac (λ 580 nm) and black (for large particles) due to a shift in the absorption peak caused by surface plasmon resonance (SPR) (Mulvaney, 1996; Huang and El-Sayed, 2010).

**Figure 1:**
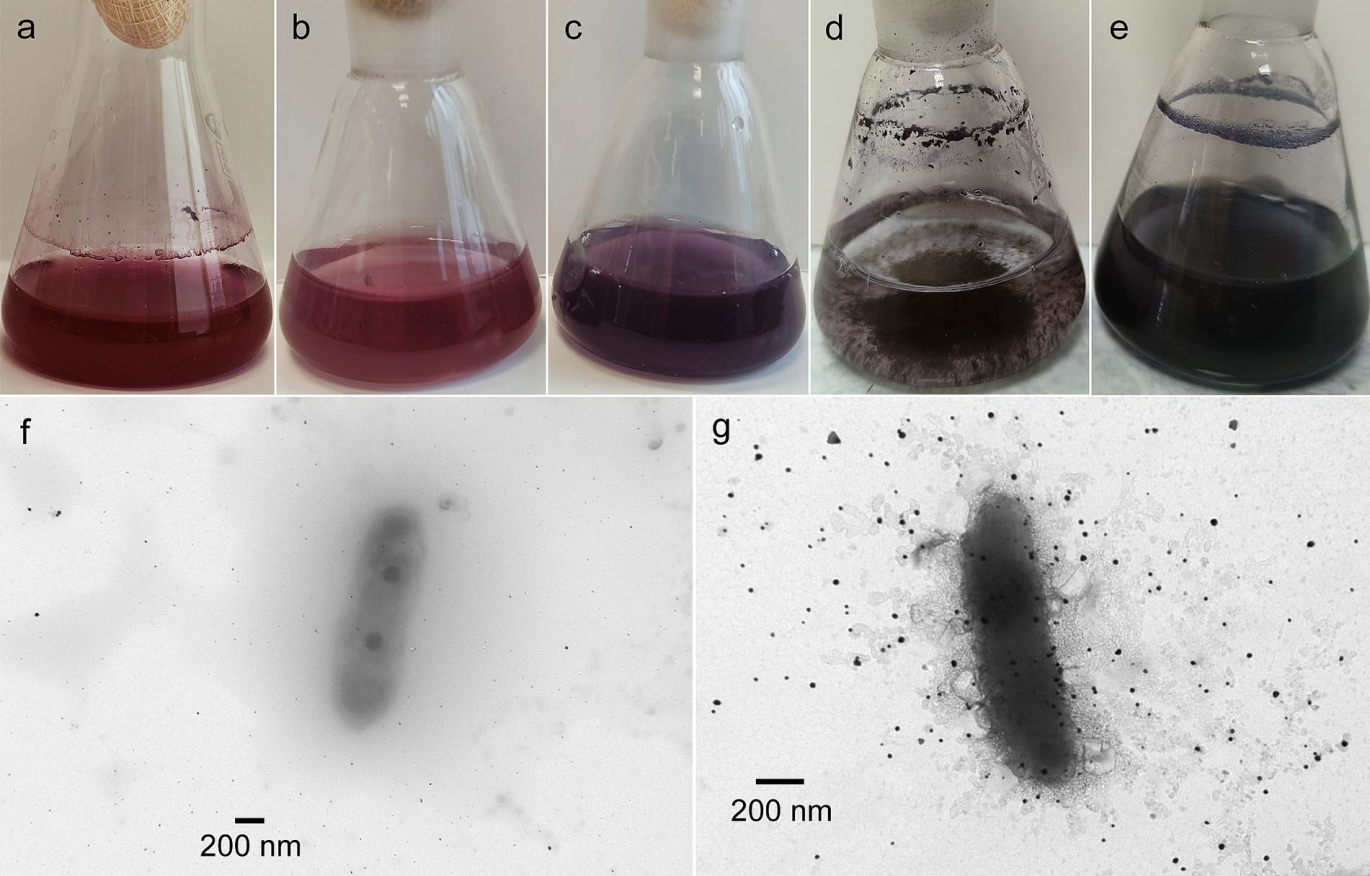
Biosynthesis of AuNPs by bacteria *Methylophilus* sp. F5P3.1 m after 6 **(a)**, 15 **(b)**, and 72 **(c)** hours, respectively; intracellular accumulation of gold by bacteria *Rhodococcus erythropolis* **(d)** and *Ancylobacter rudongensis* **(e)** 72 h after the addition of HAuCl_4_; TEM of *Methylophilus* sp. F5P3.1 m cells and AuNPs after 6 **(f)** and 15 **(g)** hours of biosynthesis, respectively.

Among the selected microbial isolates, some cultures reduced cations to form Au^0^ exclusively intracellularly. Figures 1d,e shows that very large nanoparticles or their aggregates remain bound to cells. In these cases, it was impossible to visually track the stage of formation of small nanoparticles even at the beginning of the synthesis. Sometimes, separating the nanoparticles from the biomass was difficult, even using ultrasonication and a high-speed homogenizer. After separation from the biomass, the particles remained large (>200 nm according to DLS and TEM).

We paid special attention to the study of cultures of C_1_-utilizing microorganisms that have cell-associated synthesis of AuNPs, but release these nanoparticles into the culture medium.

The most rapid synthesis and effective stabilization of AuNPs were demonstrated by the obligate methylotrophic culture designated as isolate F5P3.1 m. These fast-growing bacteria withstood dilution rate up to D = 0.42 h^−1^ in the chemostat, which exceeded the capabilities of other isolates studied (Figure 1).

**Figure 2:**
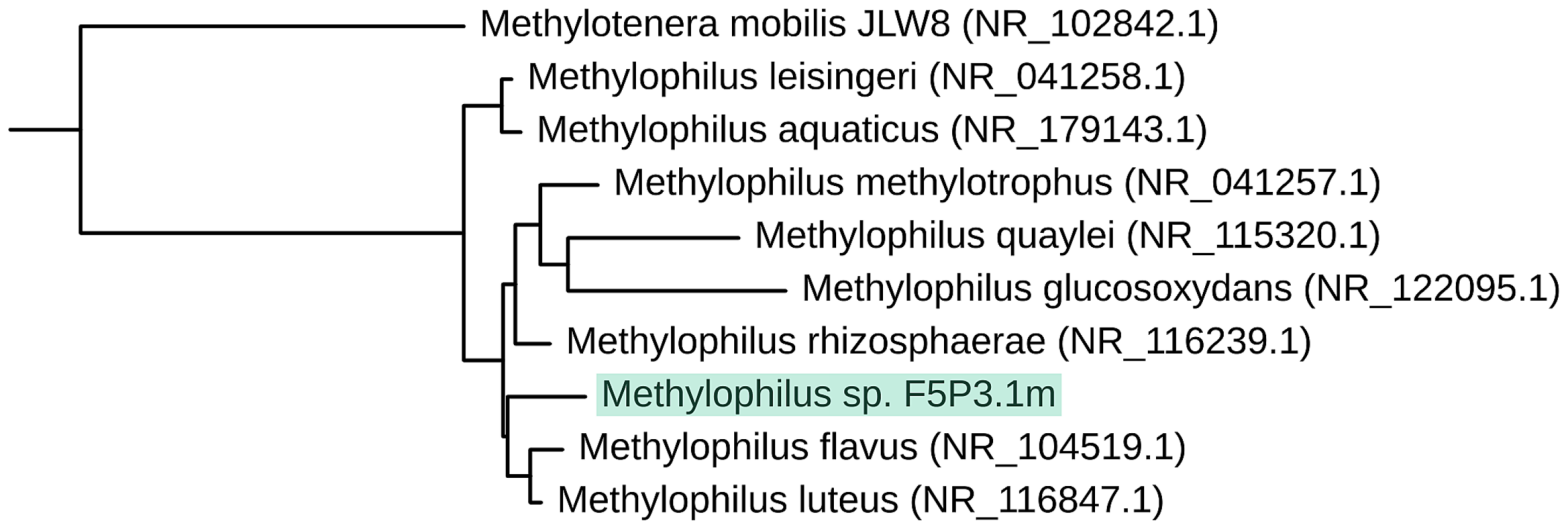
Phylogenetic tree of representatives of the genus *Methylophilus* and isolate F5P3.1 m, constructed using the neighbor-joining method.

Using 16S rRNA analysis, isolate F5P3.1 m was identified as a member of the genus *Methylophilus*. The large distance to its nearest neighbors and its distinct position on the phylogenetic tree suggest that isolate F5P3.1 m may be a potential new member of the genus *Methylophilus* (Figure 2), which requires further confirmation by other methods.

### 3.2 Biosynthesis features and characterization of obtained extracellular AuNPs

#### 3.2.1 Direct determination of nanoparticle sizes using TEM, DLS, SEM

**Figure 3:**
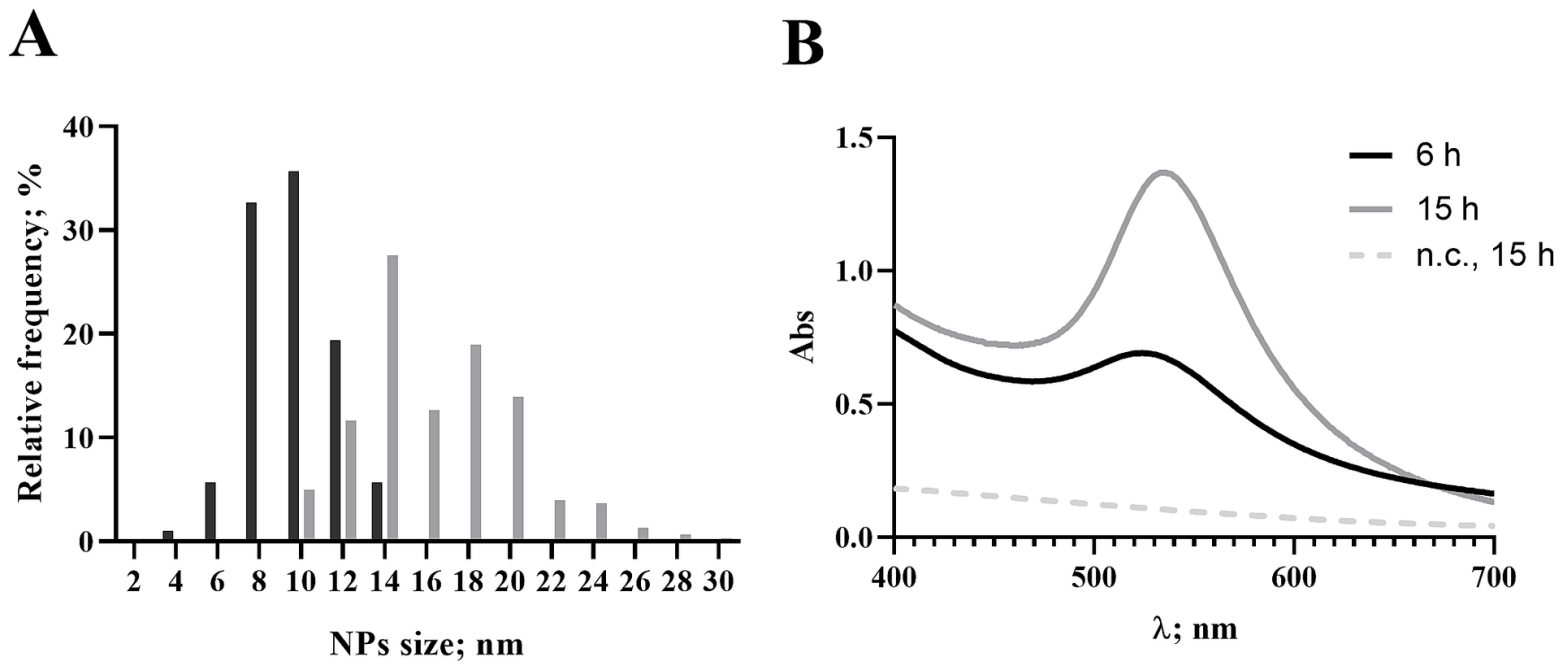
**(A)** The sizes of the gold core of nanoparticles at the 6th and 15th hours of biosynthesis, respectively; **(B)** Spectra of solutions of nanoparticles separated from biomass at the 6th and 15th hours of biosynthesis, as well as the spectrum of negative control (N. C.)—cell-free culture supernatant of cells in the active growth phase obtained by centrifugation at the 15th hour after the addition of HAuCl_4_.

Obligate methylotrophic bacteria *Methylophilus* sp. F5P3.1 m were cultivated in flasks under optimal conditions at pH = 5.5, 28 °C for 12 h. By this time, the cells were at the end of the active growth phase and over the next 15 h, with the addition of 0.45 mM HAuCl_4_, they synthesized AuNPs with predominant sizes of 9–24 nm (Figures 1g, 3A; Supplementary Figures S3, S4), which were found outside the cells in the culture medium with a 37 ± 6% yield of the initial gold content (the pH, temperature, bacterial growth phase, and HAuCl_4_ concentration were pre-optimized to maximize the yield of extracellular AuNPs).

A visual change in the cell suspension’s color to pinkish began approximately 3 h after the addition of HAuCl_4_. The peak release (approximately 15 ± 3% of the initial gold content) of the smallest nanoparticles (4–14 nm) into the culture medium occurred between 6 and 8 h (Figures 1f, 3A; Supplementary Figures S1, S2), gradually fading by 15 h of synthesis. At this time, TEM revealed intact cells surrounded by spherical, sometimes triangular, AuNPs that were not concentrated within the cell. After completion of the synthesis of small nanoparticles that were in the culture medium, gold reduction could continue intracellularly for up to 7 days with the formation of large agglomerates. A 2- or 3-day culture (inactive bacteria) of *Methylophilus* sp. F5P3.1 m bacteria synthesized reduced gold intracellularly, and the suspensions after 7 days looked like the samples in Figures 1d,e. The active synthesis of small nanoparticles, which could be isolated from the culture medium, thus coincided with the time of active metabolism of the bacterium itself.

The spectra of gold nanoparticle solutions separated from the biomass exhibited a shift in the absorption peak from *λ* 525 nm to λ 535 nm during the process of synthesis and attenuation of cellular activity (Figure 3B), which indicates the formation of larger nanoparticles closer to the end of the process. The gold core size distribution of nanoparticles, obtained from TEM data, at the beginning (6 h) and after 15 h of synthesis also shows a noticeable increase in the proportion of larger nanoparticles in the latter samples (Figure 3A). However, nanoparticle solutions collected at different times did not change their spectral characteristics for at least 2 years of storage (at concentrations up to 35 mg/L of gold), indicating their strong stabilization by the compounds coating the gold core.

Importantly, cell-free culture supernatant obtained by centrifugation under the same conditions did not synthesize nanoparticles upon addition of HAuCl_4_, as confirmed by spectral analysis (Figure 3B) and direct TEM observation. Centrifuged cells inactivated by heating to 105 °C or microwave irradiation (900 W) for 5 min and then transferred to their own supernatant also did not synthesize AuNPs upon addition of HAuCl_4_.

**Figure 4:**
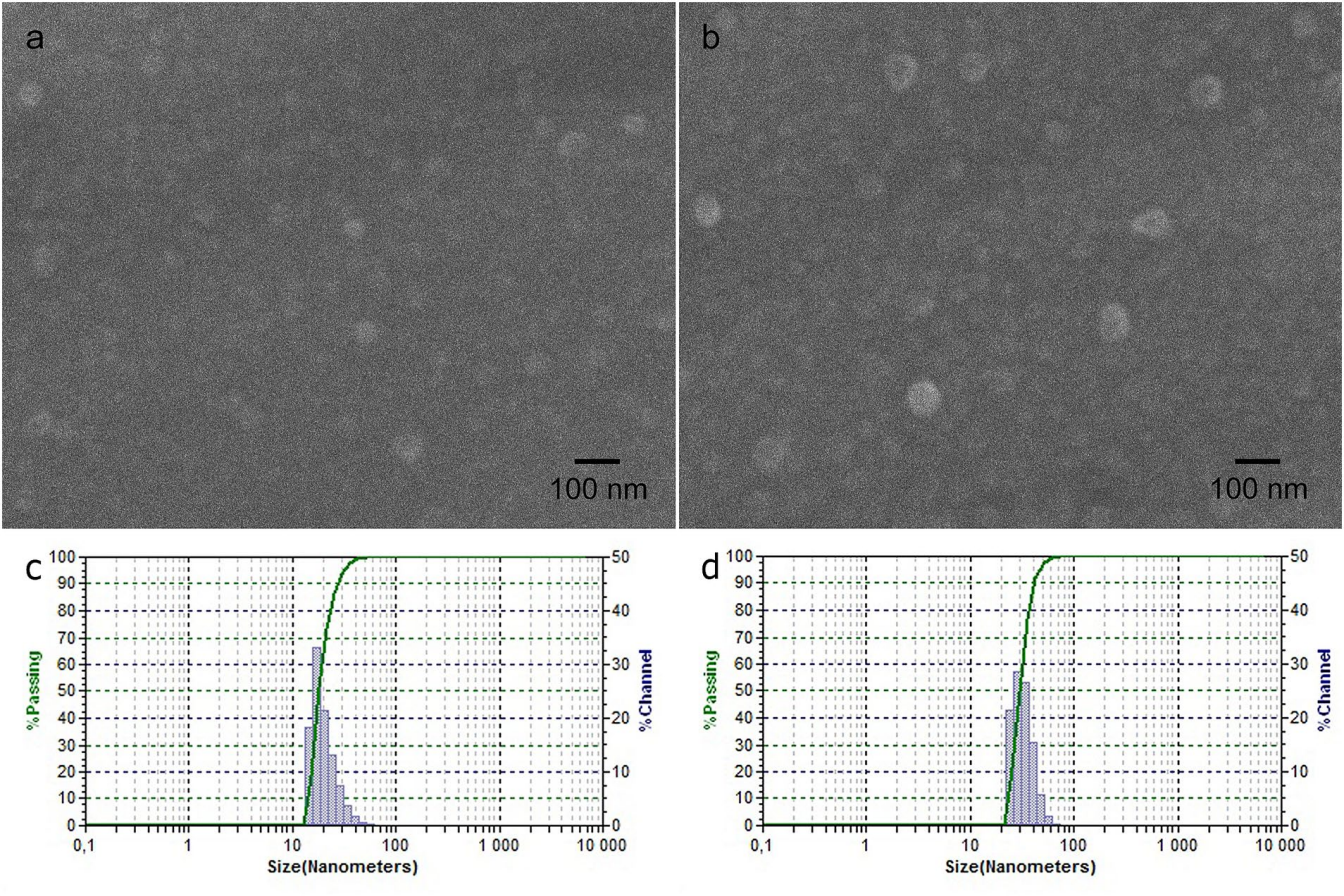
**(a,c)** SEM and DLS of shell-coated AuNPs after 6 h of synthesis, respectively; **(b,d)** SEM and DLS of shell-coated AuNPs after 15 h of synthesis, respectively.

DLS data allowed for estimation of the shell thickness covering the nanoparticle core. DLS data correlate well with direct SEM observations (Figure 4). At the beginning of the synthesis, nanoparticles with a shell and a total diameter of 14–60 nm were characteristic, and toward the end of the synthesis their sizes increased and were in the range from 22 to 70 nm.

#### 3.2.2 XRD of synthesized AuNPs

**Figure 5:**
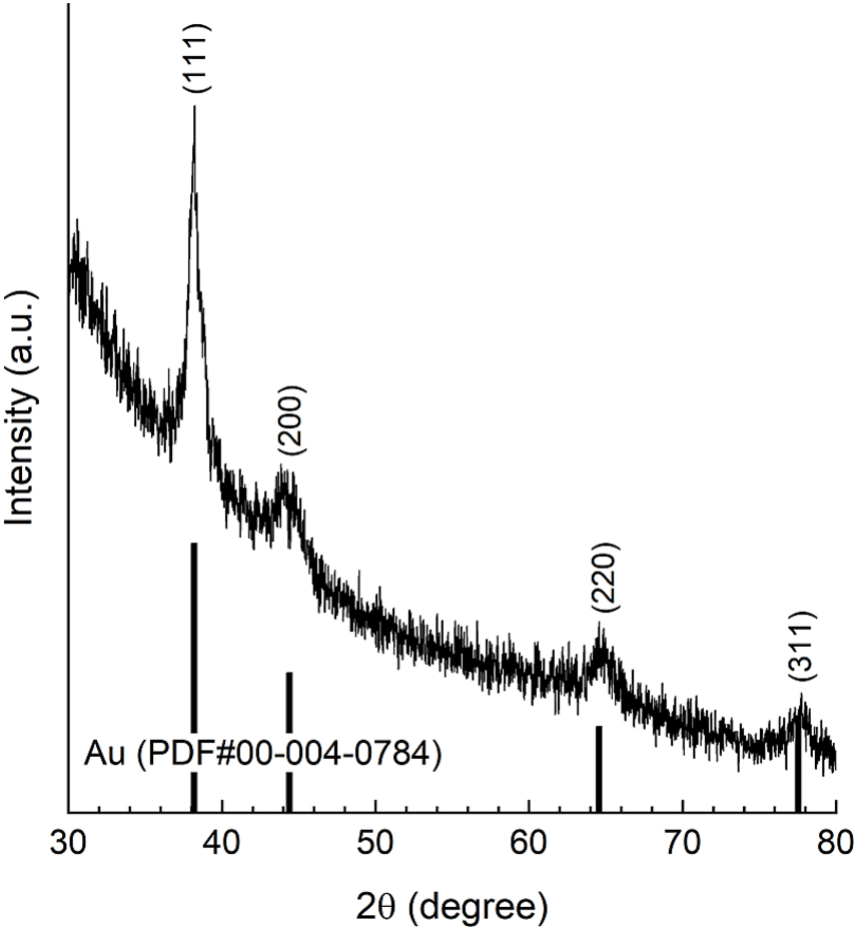
X-ray diffraction pattern of AuNPs sample after 6 h of synthesis.

The X-ray diffraction pattern of the synthesized sample shows four low-intensity peaks: 38.220°; 44.487°; 64.572°; 77.710°, the position of which, according to the PDF 4 + electronic file, corresponds to the cubic lattice of gold (PDF 00-004-0784). Moreover, the X-ray diffraction pattern shows a strong broadening of all peaks, which is typical of nanoscale materials. The coherent scattering region, calculated using the Scherrer formula, for the synthesized Au sample ranged from 4 to 10 nm (Figure 5).

#### 3.2.3 Analysis of synthesized AuNPs by FT-IR spectroscopy

**Figure 6:**
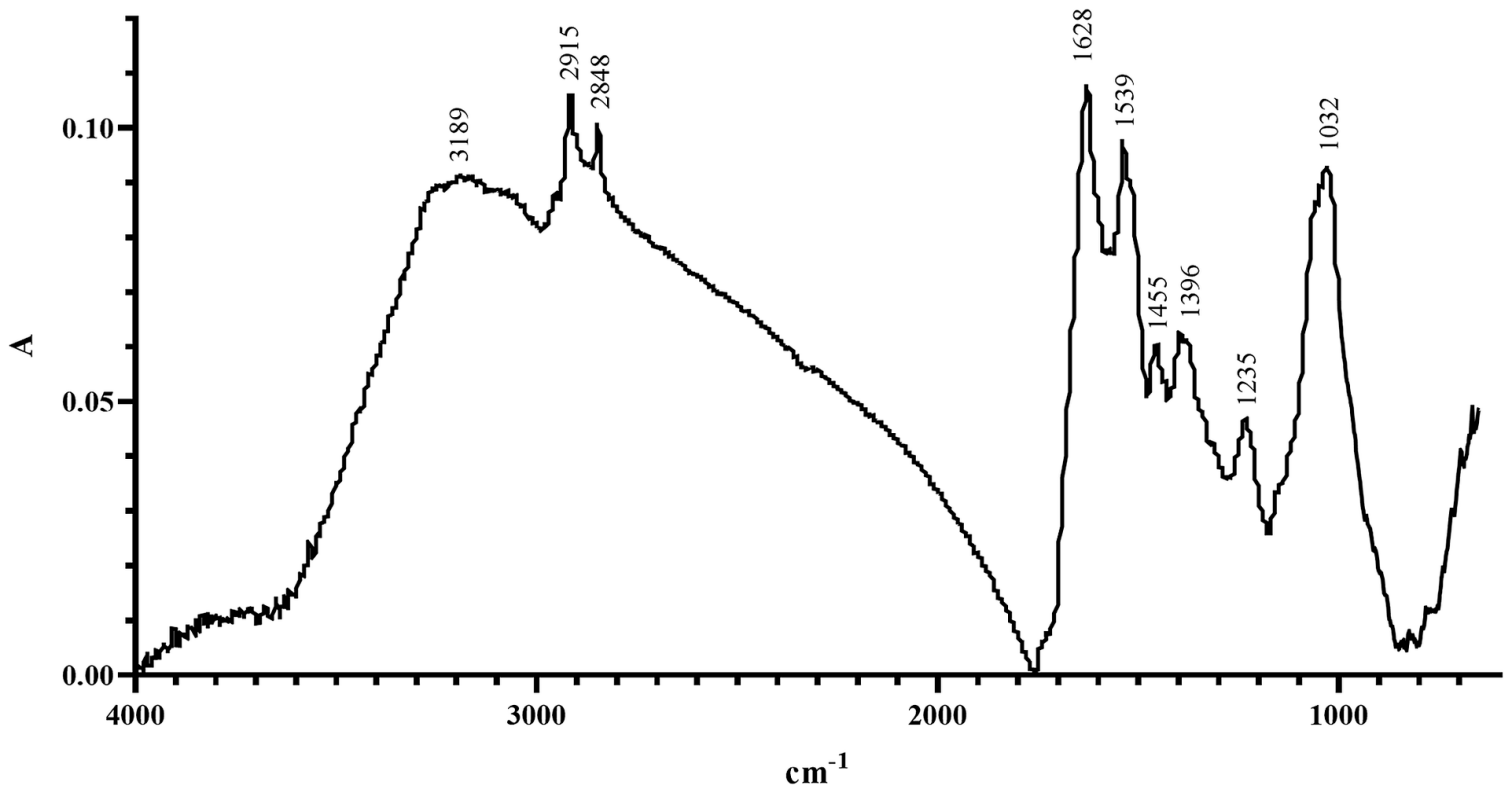
FT-IR spectra of of AuNPs sample after 6 h of synthesis.

The peptide bond of proteins is of amide nature and has characteristic ranges of absorption of infrared (IR) radiation: the region of amide I (1,600–1,800 cm^−1^), amide II (1,470–1,570 cm^−1^), amide III (1,250–1,350 cm^−1^) and amide A and amide B (~3,300 cm^−1^ and ~3,100 cm^−1^, respectively) (Tatulian, 2012; Myshakina and Asher, 2007; Ji et al., 2020). The boundaries of the given spectral regions are approximate and strongly depend on both the nature of the analyzed material (composition, spatial structure and state of the protein) and on the technical features of obtaining a specific IR spectrum (Tatulian, 2012). The IR spectrum of the AuNPs synthesized by *Methylophilus* sp. F5P3.1 m bacteria, shown in Figure 6, has distinct bands with absorption maxima characterized by wavelengths of 1,234 cm^−1^, 1,455 cm^−1^, 1,538 cm^−1^, 1,628 cm^−1^, and 3,189 cm^−1^, corresponding to the above-mentioned absorption ranges of the amide bond.

Various X–H groups, where X is C, N or O, absorb in the region of 2,800–3,000 cm^−1^, characterized by the presence of two pronounced absorption peaks: 2,848 and 2,915 cm^−1^, and can also make a certain contribution to the absorption in the region of amide A and amide B. Examples of such groups include the C–H bonds of the CH_2_ groups of alkanes (2,840–3,000 cm^−1^), aromatic compounds (3,030–3,080 cm^−1^), N–H groups of amines (in the ammonium form in the range of 2,000–3,000 cm^−1^ they give a broad band of medium intensity), O–H bonds that are part of the carboxyl groups of carboxylic acids (3,550–2,500 cm^−1^). All of the above groups are found in the side chains of proteinogenic amino acids. Also, water molecules absorb in this range (Tatulian, 2012).

The intense absorption peak at 1,396 cm^−1^ together with the peak at 1,538 cm^−1^ may correspond to carboxyl groups whose oxygen atoms coordinate the metal atom. Carboxyl groups in this state are manifested by two intense absorption peaks in the ranges of 1,650–1,540 cm^−1^ and 1,450–1,360 cm^−1^ (Smith, 2018; El-Deeb et al., 2022). The peak at 1628 cm^−1^ may also correspond to carboxyl groups bound to the surface of AuNPs (Khalkho et al., 2022).

The absorption peak at 1396 cm^−1^ may also correspond to deformations of the C-H bonds of CH_3_ and CH_2_ groups.

The absorption peak 1031.5 cm^−1^ in the range of 950–1,150 cm^−1^ may correspond to deformation vibrations of C–C (1,016 cm^−1^) and C–N (1,080 cm^−1^) groups of amino acid side radicals (Ridgley et al., 2013), C–N bonds of aromatic amines, as well as S=O bonds of sulfur-containing functional groups formed from -SH groups as a result of oxidation by Au^(+3/+1)^ (El-Deeb et al., 2022; Cerra et al., 2021).

#### 3.2.4 Electrophoresis of proteins that stabilize gold nanoparticles

**Figure 7:**
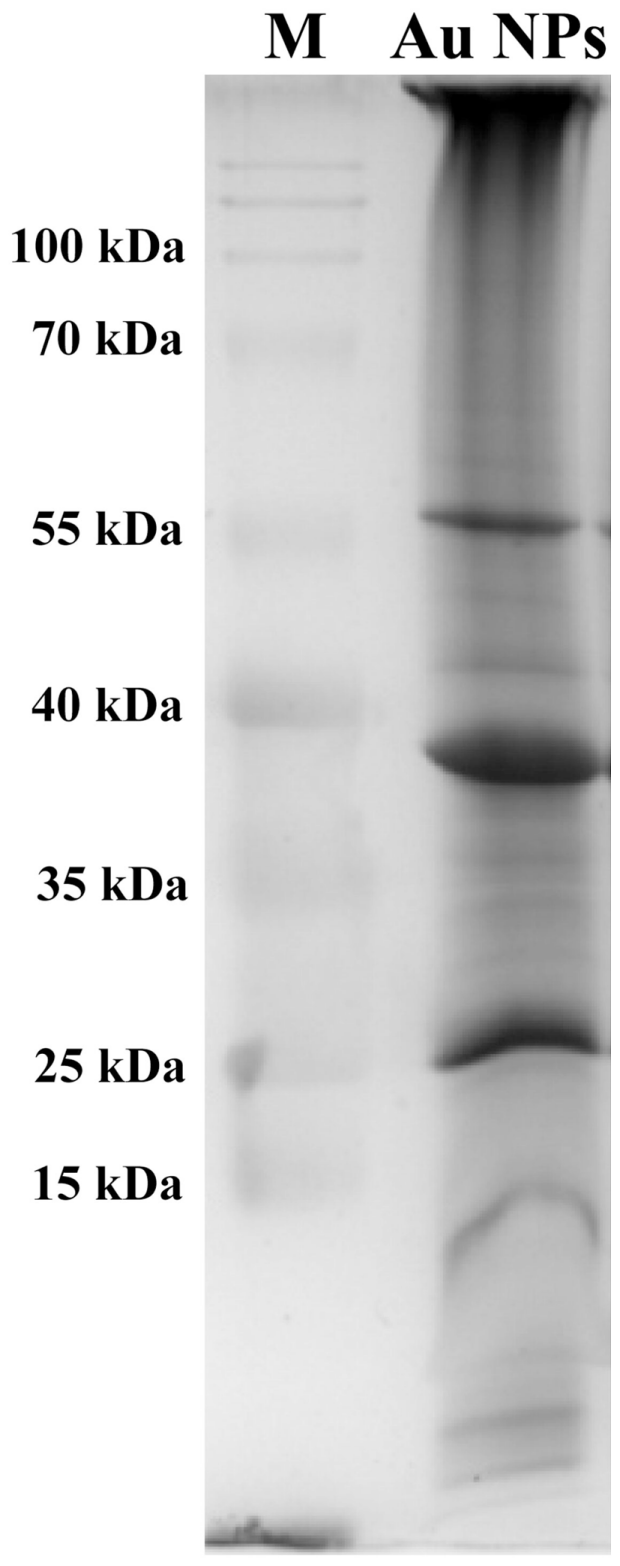
Electropherogram of the proteins of the AuNPs shell.

The electropherogram of AuNPs shell proteins shows 8 bands, which correspond to proteins with molecular masses of 55.5 (i), 48 (ii), 43 (iii), 36 (iv), 32.5 (v), 31 (vi), 28 (vii) and 24 (viii) kDa (Figure 7). Based on the intensity of the band color, it can be concluded that the most represented components of the shell of the studied AuNPs are proteins with molecular masses of 36, 24 and 55.5 kDa. Additionally, mass spectrometric analysis of the protein composition of the AuNPs shell was carried out. We also studied the protein shell of AgNPs produced *Methylophilus* F5P3.1 m (unpublished data) using SDS-PAGE electrophoresis and mass spectrometry. The data obtained by both research methods indicate the similarity of the protein compositions of the shells of AgNPs and AuNPs. According to the results of SDS-PAGE electrophoresis, the shell of AgNPs includes at least 15 proteins, 5 of which, including the 3 most abundant, have molecular masses comparable to the molecular masses of proteins found in the shell of AuNPs (55.5, 43, 36, 31, and 24 kDa). Based on the results of proteomic analysis of the AuNPs shell, four of these five proteins were identified as unidentified porin (43 kDa), TorF family putative porin (36 kDa), flagellin (31 kDa), and OmpW family outer membrane protein (24 kDa).

#### 3.2.5 Biocidal properties of gold nanoparticles obtained using methylotrophic bacteria

**Figure 8:**
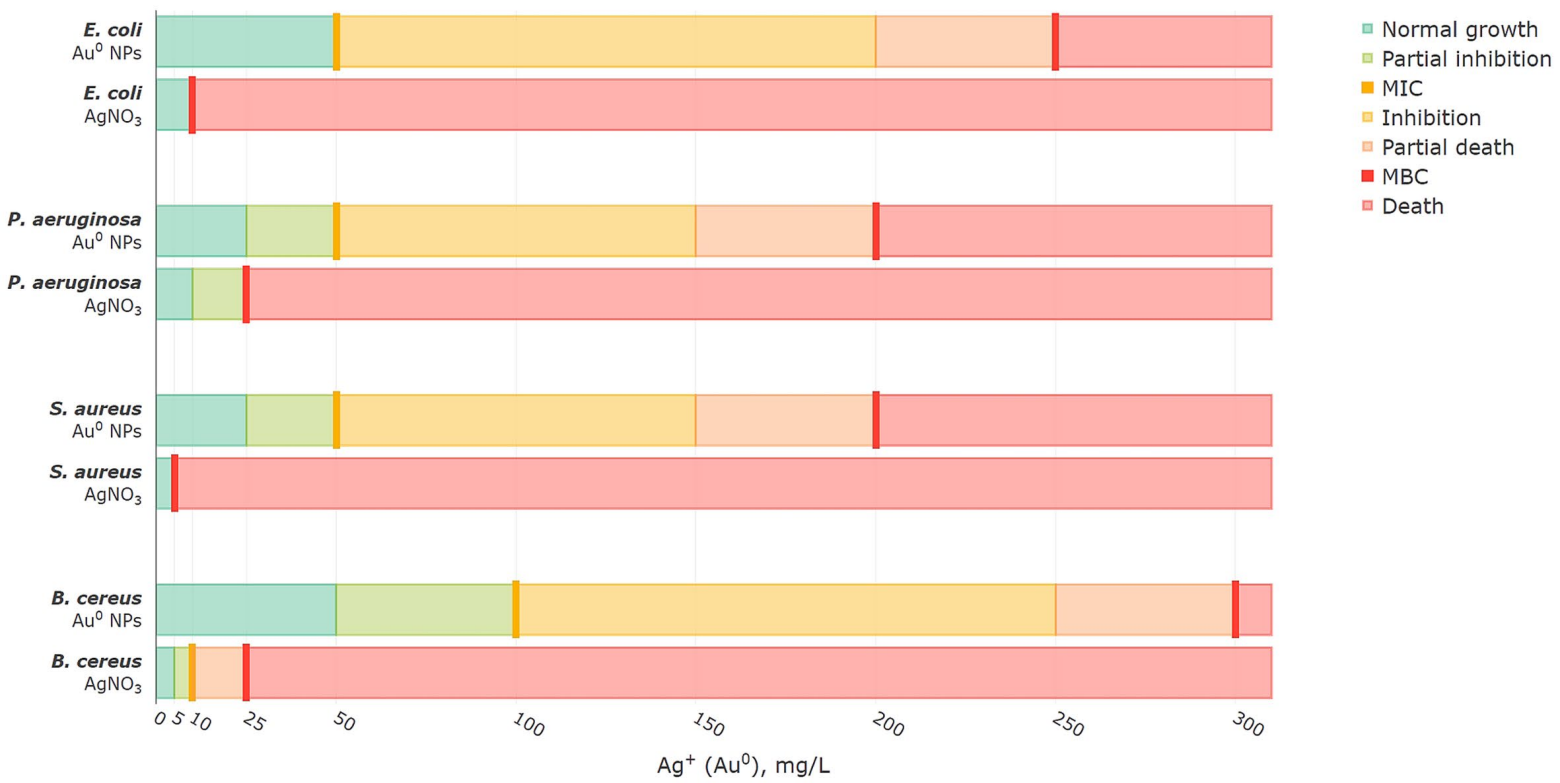
Comparison of the survival rate of pathogenic microorganisms when exposed to different concentrations of AuNPs and AgNO_3_.

Based on the obtained results of the antibacterial activity assessment, AuNPs are characterized by significantly weaker biocidal properties in comparison with AgNO_3_. The MBC of AuNPs varies in the range of 200–300 mg/L, while complete inactivation of test cultures of pathogens by AgNO_3_ was observed already at 5–25 mg Ag^+^/L. However, it is important to note the pronounced bacteriostatic properties of AuNPs. The MIC of AuNPs was 50 mg/L against *E. coli*, *P. aeruginosa* and *S. aureus* and 100 mg/L against *B. cereus* (Figure 8).

#### 3.2.6 Antioxidant activity of AuNPs coated with an organic stabilizing shell

**Figure 9:**
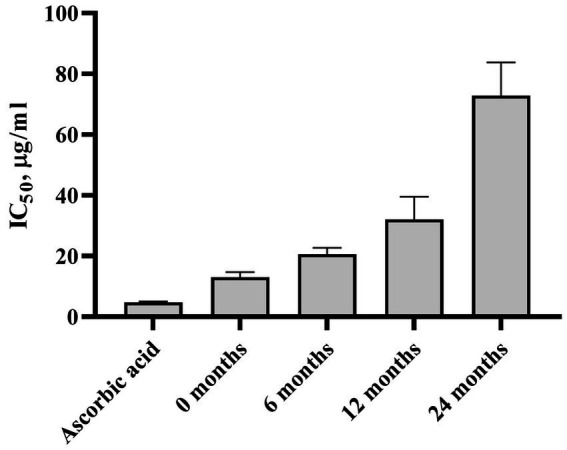
Influence of storage time on the antioxidant activity of AuNPs.

The synthesized gold nanoparticles exhibit high antioxidant activity in scavenging free radicals against DPPH, which is mediated by the bio-derived shell surrounding the gold core. This shell is likely composed of bacterial cell membrane components. Initially, the high antiradical activity is ensured by the synergism between the catalytically active gold surface and the abundance of hydrogen and electron donors within this biogenic coating. The IC_50_ value for the as-synthesized AuNPs is 13.0 ± 1.7 μg/mL, compared to 4.75 ± 0.25 μg/mL for ascorbic acid. During long-term storage of AuNPs in the dark at 4 °C, the IC_50_ increased continuously throughout the entire observation period. After the first 6 months of storage, it did not change significantly, remaining at 20.6 ± 2.1 μg/mL. However, upon further exposure, the value reached 32.0 ± 7.5 μg/mL after 1 year and amounted to 72.9 ± 10.9 μg/mL after 2 years of storage. Since the IC_50_ value is inversely proportional to the antioxidant capacity, this trend indicates a drop in the radical scavenging capacity of the nanoparticles by approximately 58% in the first 6 months, by 2.5 times by the end of the first year, and by more than 5.6 times by the end of the second year of storage (Figure 9).

The antioxidant activity of gold nanoparticles synthesized using microorganisms varies over a wide range. Specifically, AuNPs synthesized extracellularly at 45 °C using a cell-free supernatant obtained from *Lysinibacillus odysseyi* culture exhibited an IC_50_ value of less than 20 μg/mL (Cherian et al., 2022). In contrast, nanoparticles synthesized intracellularly by bacteria of the same genus at 37 °C demonstrated an IC_50_ of 233.75 μg/mL (Markus et al., 2016). For nanoparticles synthesized using the cell-free extract of *Paracoccus haeundaensis* at 70 °C, the IC_50_ value was approximately 80 μg/mL (Patil et al., 2019). These findings clearly demonstrate the decisive role of the capping shell composition in the antioxidant activity of microbially synthesized AuNPs. The high antioxidant activity of AuNPs depends on the synergy arising from the interaction between the gold core and its capping compounds (Suliasih et al., 2024). The physicochemistry and biochemistry underlying these processes warrant comprehensive further investigation.

## 4 Discussion

In the literature, the consideration of issues of generation of metal nanoparticles in the presence of microorganisms is often combined with studies of the cellular response to stress caused primarily by heavy metals, copper and silver ions (Nies, 2016; Bütof et al., 2018; Nnaji et al., 2024). In accordance with the classical works of Gadd (2010), describing the synthesis of metal nanoparticles using microorganisms, the localization of synthesis is specified—“extracellular” or “intracellular.” In cases of cell-associated synthesis of nanoparticles, researchers talk about the usually involved active processes of biosynthesis and secretion of certain reducing equivalents, transport processes and interactions with surface biopolymers. In the present study, no AuNP synthesis was observed in culture fluid separated from cells under physiological conditions and with the addition of HAuCl_4_. It would be more accurate to speak of cell-associated AuNP synthesis.

Since this study observed a correlation between the level of gold nanoparticle generation and the state of the producer culture, we will examine in more detail the physiological responses of microorganism cells to the presence of cations in the growth medium.

The literature provides detailed descriptions of stress response systems to heavy metal, and the most studied are the mechanisms of copper detoxification (Nies, 2016; Bütof et al., 2018). So-called non-essential metals, which include gold, have received insufficient attention in the literature. However, three levels/biosystems can be identified that provide protection to living cells from the toxic effects of gold ions.

The first level of protection includes chemisorption processes that can occur between gold ions and protein molecules, polysaccharides, and other metabolites present in the cell environment. Capsules and outer surface structures of the cell, S-layer proteins are also often involved in these processes (Ghosh et al., 2021; Singh et al., 2024; Campaña et al., 2023).

The second level of detoxification involves the reduction of gold by sulfur-containing amino acid residues of outer membrane proteins, namely methionine and cysteine. The possible involvement of Cop proteins, rich in methionine and cysteine, which are found in the periplasm (CopA, CopK, CopJ, CopC) and outer membrane (CopB) in AuNPs synthesis in *C. metallidurans* bacteria has been described (Montero-Silva et al., 2017).

Active transport systems are the third and critical level in bacterial cells’ fight against heavy metal ion toxicity. Regulation of these biosystems is closely linked to metal ion concentrations, allowing bacteria to adapt to changing conditions. Copper transport systems, particularly the CupA ATPase and Cus system, have also been thoroughly studied in *C. metallidurans*. One component of this copper-dependent system is CopA, which, as shown in (Bütof et al., 2018), promotes the survival of *C. metallidurans* in gold-rich environments by converting it into nanoparticles localized in the periplasm. Thus, highly specific detoxification processes for one metal may also contribute to a non-specific process for gold ions.

All of the above does not fully explain the extracellular localization of AuNPs in the culture medium and the increase in their concentration in the presence of active living cells. One day after adding 0.45 mM HAuCl_4_, the number of dead cells increases by only 8–15%, depending on the series of experiments. Even 3 days after adding HAuCl_4_, approximately 70–80% of the cells remain viable (approximately the same as in the control sample), although the cells themselves are now stained lilac-black, indicating intracellular gold reduction. Thus, the chosen concentration of HAuCl_4_ is, of course, stressful, but sublethal for *Methylophilus* sp. F5P3.1 m.

We propose as a working hypothesis (requires further confirmation) that stress caused by gold ions plays a key role in the formation of nanoparticles coated with a peculiar shell. It triggers the formation of bacterial extracellular vesicles (BEVs) or membrane vesicles. According to (Nnaji et al., 2024; Mozaheb and Mingeot-Leclercq, 2020; Caruana and Walper, 2020), vesicle production is a universal bacterial defense response to various stressors, including antibiotics, heavy metals, and oxidative stress. Vesicles are formed by cleavage from the outer membrane (OMV), and AuNPs are “packaged” into the vesicles for exit from the cell, preventing their accumulation inside. The shell of porins (Prajapati et al., 2021) and flagellin (Wen et al., 2023) suggests that AuNPs are formed in the periplasm and released externally through budding OMVs, reducing AuNPs toxicity. This process resembles the spontaneous formation of biomimetic nanoparticles, albeit without additional steps (Naskar et al., 2021).

## 5 Conclusion

C_1_-utilizing bacteria are important industrial biotechnological producers, widely used to obtain microbial protein and valuable bioactive compounds. However, their biosynthetic potential has not yet been realized in nanobiotechnology. This study was conducted to isolate methylotrophic bacteria specifically for their subsequent use as producers of nanoparticles, particularly gold.

The paper presents the results of the isolation of new methylotrophic bacteria in pure form, characterized by high growth characteristics when cultivated on a mineral growth medium with methanol as the sole source of carbon and energy. Among the many methylotrophic cultures, obligate methylotrophic bacteria of the genus *Methylophilus* were selected, which have the highest reducing capacity for gold cations. The features of biosynthesis and dynamics of gold nanoparticle generation were studied in detail, the features of АuNPs localization at various stages of the producer bacterium growth were studied, and the possibilities of regulation for the synthesis of extracellular gold nanoparticles with certain parameters were shown. It is important to emphasize that the use of obligate methylotrophic bacteria, which do not require the presence of complex organic substrates in the nutrient medium for growth, in principle makes it possible to control/manage the biosynthesis of NPs stabilizers, which is extremely important for the production of their monodisperse preparations. Moreover, since genetic engineering of *Methylophilus* bacteria is well developed, it is possible to specifically functionalize the generated AuNPs directly during their *de novo* formation. Overall, the study demonstrated that selected obligate methylotrophic bacteria of the genus *Methylophilus* could become a promising high-yield microbial producer for green biotechnological synthesis of target metal nanoparticles.

## Data Availability

The original contributions presented in the study are included in the article/supplementary material and are also publicly available. These data can be found: https://www.ncbi.nlm.nih.gov/nuccore/PZ476803; https://www.ncbi.nlm.nih.gov/nuccore/PZ476804; https://www.ncbi.nlm.nih.gov/nuccore/PZ476805.

## Footnotes

1
http://www.ncbi.nlm.nih.gov/blast
