# Supplementary material for: Active biosynthesis of gold nanoparticles mediated by obligate methylotrophic bacteria Methylophilus sp
Source: Front Microbiol. 2026 Jun 12;17:1852773. doi: 10.3389/fmicb.2026.1852773 (PMC13303613; doi:10.3389/fmicb.2026.1852773)
Supplement: Supplementary file 1 [file Data_Sheet_1.docx]

Supplementary Material

# Supplementary Data

**16S rRNA sequences of microorganisms presented in the paper (sequences will be added to GenBank later with reference to the published paper).**

>*Methylophilus* sp. F5P3.1m

ACCATGCAGTCGAACGATGAACCCTAGCTTGCTAGGGGAATTAGTGGCGAACGGGTGAGTAATATATCGGAACGTGCCTTGTAATGGGGGATAACTAGTCGAAAGATTAGCTAATACCGCATACGCCCTGAGGGGGAAAGTAGGGGATCTTCGGACCTTACGTTATAAGAGCGGCCGATATCTGATTAGCTAGTTGGTGGGGTAATGGCCTACCAAGGCGACGATCAGTAGCTGGTCTGAGAGGACGACCAGCCACACTGGAACTGAGACACGGTCCAGACTCCTACGGGAGGCAGCAGTGGGGAATTTTGGACAATGGGCGAAAGCCTGATCCAGCCATTCCGCGTGAGTGAAGAAGGCCTTCGGGTTGTAAAGCTCTTTCGCAAGGGAAGAAAACTTACATTCTAATAAAGTGTGAGGCTGACGGTACCTTGATAAGAAGCACCGGCTAACTACGTGCCAGCAGCCGCGGTAATACGTAGGGTGCGAGCGTTAATCGGAATTACTGGGCGTAAAGCGTGCGCAGGCGGTTTGGCAAGTCAGATGTGAAATCCCCGAGCTCAACTTGGGAACTGCGTTTGAAACTGCCAGACTAGAATATGTCAGAGGGGGGTAGAATTCCACGTGTAGCAGTGAAATGCGTAGAGATGTGGAGGAATACCAATGGCGAAGGCAGCCCCCTGGGATAATATTGACGCTCATGCACGAAAGCGTGGGGAGCAAACAGGATTAGATACCCTGGTAGTCCACGCCCTAAACGATGTCTACTAGTTGTTGGTGGAGTAAAATCCATGAGTAACGCAGCTAACGCGTGAAGTAGACCGCCTGGGGAGTACGGTCGCAAGATTAAAACTCAAAGGAATTGACGGGGGCCCGCACAAGCGGTGGATTATGTGGATTAATTCGATGCAACGCGAAAAACCTTACCTGGCCTTGACATGCCACTAACGAAGCAGAGATGCATTAGGTGCCCGTAAGGGAAAGTGGACACAGGTGCTGCATGGCTGTCGTCAGCTCGTGTCGTGAGATGTTGGGTTAAGTCCCGCAACGAGCGCAACCCTTGCCATTAATTGCCATCATTTAGTTGGGCACTTTAATGGGACTGCCGGTGACAAACCGGAGGAAGGTGGGGATGACGTCAAGTCCTCATGGCCCTTATGGCCAGGGCTTCACACGTAATACAATGGTCGGTACAGAGAGTTGCCAACCCGCGAGGGGGAGCTAATCTCAGAAAGCCGATCGTAGTCCGGATTGTTCTCTGCAACTCGAGAGCATGAAGTCAGAATCGCTAGTAATCGCGGATCAGCATGTCGCGGTGAATACGTTCCCGGGCCTTGTACACACCGCCCGTCACACCATGGGAGTGGGTTTTACCAGAAGTAGTTAGTCTAACCGCAAGGAGGACGATTACCACGGTAGTATTCATGACTGGGGTGAAGTCGT

>*Rhodococcus erythropolis*

ACAATTGCCGGCATGCTTACCATGCAGTCGAGCGGTAAGGCCTTTCGGGGTACACGAGCGGCGAACGGGTGAGTAACACGTGGGTGATCTGCCCTGCACTTCGGGATAAGCCTGGGAAACTGGGTCTAATACCGGATATGACCTCAGGTTGCATGACTTGGGGTGGAAAGATTTATCGGTGCAGGATGGGCCCGCGGCCTATCAGCTTGTTGGTGGGGTAATGGCCTACCAAGGCGACGACGGGTAGCCGACCTGAGAGGGTGACCGGCCACACTGGGACTGAGACACGGCCCAGACTCCTACGGGAGGCAGCAGTGGGGAATATTGCACAATGGGCGAAAGCCTGATGCAGCGACGCCGCGTGAGGGATGACGGCCTTCGGGTTGTAAACCTCTTTCAGCAGGGACGAAGCGCAAGTGACGGTACCTGCAGAAGAAGCACCGGCTAACTACGTGCCAGCAGCCGCGGTAATACGTAGGGTGCAAGCGTTGTCCGGAATTACTGGGCGTAAAGAGTTCGTAGGCGGTTTGTCGCGTCGTTTGTGAAAACCAGCAGCTCAACTGCTGGCTTGCAGGCGATACGGGCAGACTTGAGTACTGCAGGGGAGACTGGAATTCCTGGTGTAGCGGTGAAATGCGCAGATATCAGGAGGAACACCGGTGGCGAAGGCGGGTCTCTGGGCAGTAACTGACGCTGAGGAACGAAAGCGTGGGTAGCGAACAGGATTAGATACCCTGGTAGTCCACGCCGTAAACGGTGGGCGCTAGGTGTGGGTTCCTTCCACGGAATCCGTGCCGTAGCTAACGCATTAAGCGCCCCGCCTGGGGAGTACGGCCGCAAGGCTAAAACTCAAAGGAATTGACGGGGGCCCGCACAAGCGGCGGAGCATGTGGATTAATTCGATGCAACGCGAAGAACCTTACCTGGGTTTGACATATACCGGAAAGCTGCAGAGATGTGGCCCCCCTTGTGGTCGGTATACAGGTGGTGCATGGCTGTCGTCAGCTCGTGTCGTGAGATGTTGGGTTAAGTCCCGCAACGAGCGCAACCTCTATCTTATGTTGCCAGCACGTTATGGTGGGGACTCGTAAGAGACTGCCGGGGTCAACTCGGAGGAAGGTGGGGACGACGTCAAGTCATCATGCCCCTTATGTCCAGGGCTGCACACATGCTACAATGGCCAGTACAGAGGGCTGCGAGACCGTGATGTGTAGCGAATCTCTTAAAGCTGGTCTCAGTTCGGATCGGGGTCTGCAACTCGACCCCGTGAAGTCGGAGTCGCTAGTAATCGCAGATCAGCAACGCTGCGGTGAATACGTTCCCGGGCCTTGTACACACCGCCCGTCACGTCATGAAAGTCGGTAACACCCGAAGCCGGTGGCTTAACCCCTTGTGGGAGGGAGCCGTCGAAGGTGGGATCGGCGATTGGGACGAAGTCGTAACAAGGTA

>*Ancylobacter rudongensis*

ACGCTCAGAACGAACGCTGGCGGCAGGCTTAACACATGCAAGTCGAACGCCCCGCAAGGGGAGTGGCAGACGGGTGAGTAACACGTGGGGATCTGCCCAATGGTACGGAATAATTCCGGGAAACTGGGACTAATACCGTATGTGCCCGCAAGGGGAAAGATTTATCGCCATTGGATGAACCCGCGTCGGATTAGCTAGTTGGTGAGGTAAAGGCTCACCAAGGCGACGATCCGTAGCTGGTCTGAGAGGATGATCAGCCACACTGGGACTGAGACACGGCCCAGACTCCTACGGGAGGCAGCAGTGGGGAATATTGGACAATGGGCGCAAGCCTGATCCAGCCATGCCGCGTGAGTGATGAAGGCCTTAGGGTTGTAAAGCTCTTTCGCCGACGAAGATAATGACGGTAGTCGGAGAAGAAGCCCCGGCTAACTTCGTGCCAGCAGCCGCGGTAATACGAAGGGGGCTAGCGTTGTTCGGAATCACTGGGCGTAAAGCGCACGTAGGCGGACATTTAAGTCAGGGGTGAAAGCCTGGAGCTCAACTCCAGAACTGCCCTTGATACTGGGTGTCTCGAGTCCGGAAGAGGTAAGTGGAACTGCGAGTGTAGAGGTGAAATTCGTAGATATTCGCAAGAACACCAGTGGCGAAGGCGGCTTACTGGTCCGGTACTGACGCTGAGGTGCGAAAGCGTGGGGAGCAAACAGGATTAGATACCCTGGTAGTCCACGCCGTAAACGATGGAGGCTAGCCGTTGGTGAGCATGCTCATCAGTGGCGCAGCTAACGCATTAAGCCTCCCGCCTGGGGAGTACGGTCGCAAGATTAAAACTCAAAGGAATTGACGGGGGCCCGCACAAGCGGTGGAGCATGTGGTTTAATTCGAAGCAACGCGCAGAACCTTACCAGCCTTTGACATGTCCCGGACGGTTACCAGAGATGGTTTCTTCTCTTCGGAGCCGGGAACACAGGTGCTGCATGGCTGTCGTCAGCTCGTGTCGTGAGATGTTGGGTTAAGTCCCGCAACGAGCGCAACCCTCGCCCTTAGTTGCCATCATTCAGTTGGGCACTCTAGGGGGACTGCCGGTGATAAGCCGAGAGGAAGGTGGGGATGACGTCAAGTCCTCATGGCCCTTACGGGCTGGGCTACACACGTGCTACAATGGCGGTGACAGTGGGAAGCGAACCCGCGAGGGTAAGCAAATCTCCAAAAGCCGTCTCAGTTCGGATTGCACTCTGCAACTCGAGTGCATGAAGTTGGAATCGCTAGTAATCGTGGATCAGCATGCCACGGTGAATACGTTCCCGGGCCTTGTACACACCGCCCGTCACACCATGGGAGTTGGTTTTACCCGAAGGCGCTGCGCTAACCCGCAAGGGAGGCAGGCGACCACGGTAGGGTCAGCGACTGGGGTGAAGTCGTAACAAGGTTAACCGTAA

# Supplementary Figures and Tables

## Supplementary Figures


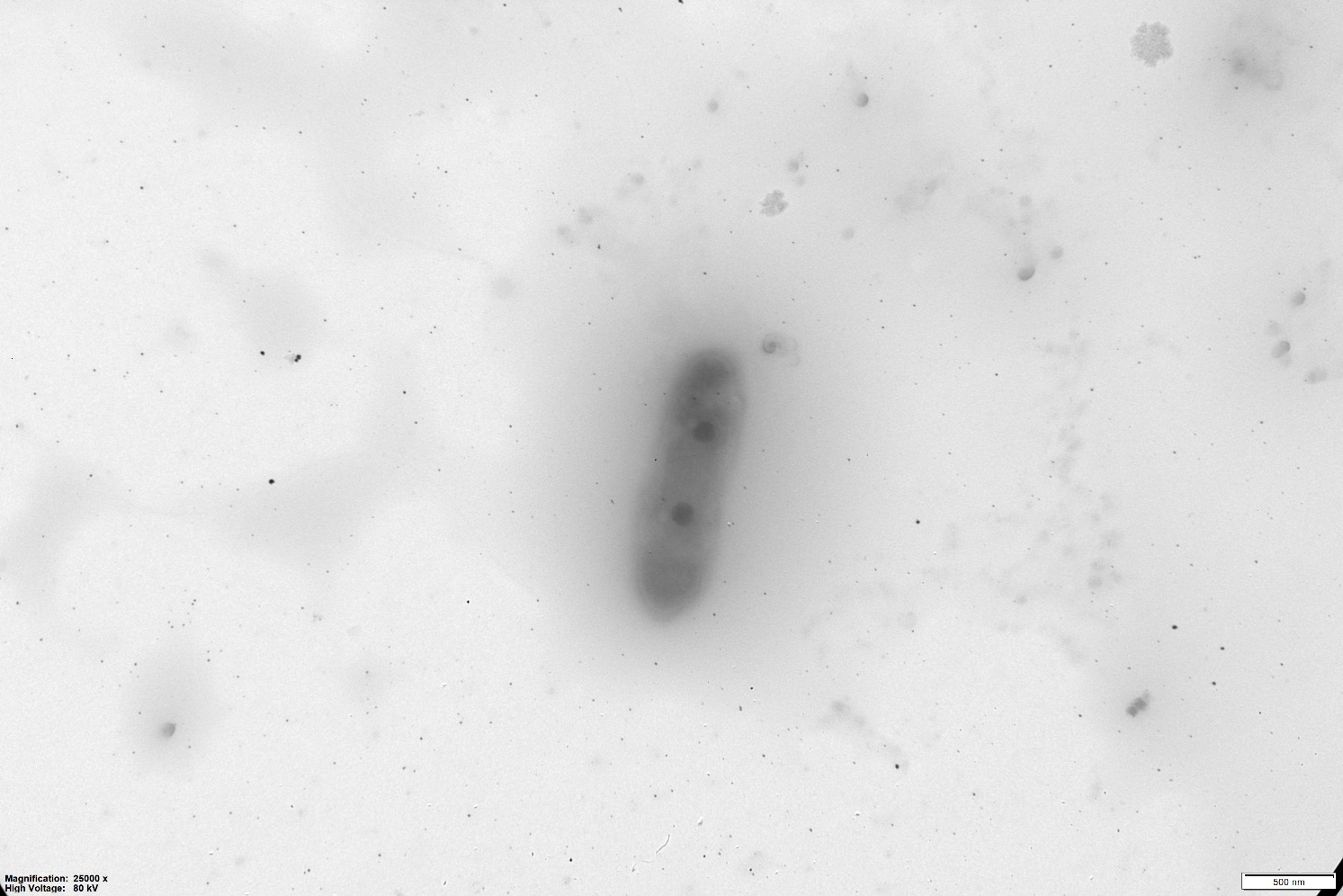


**Supplementary Figure S1**. Original TEM image of *Methylophilus* sp. F5P3.1m cells and AuNPs after 6 hours of biosynthesis with a larger field of view.


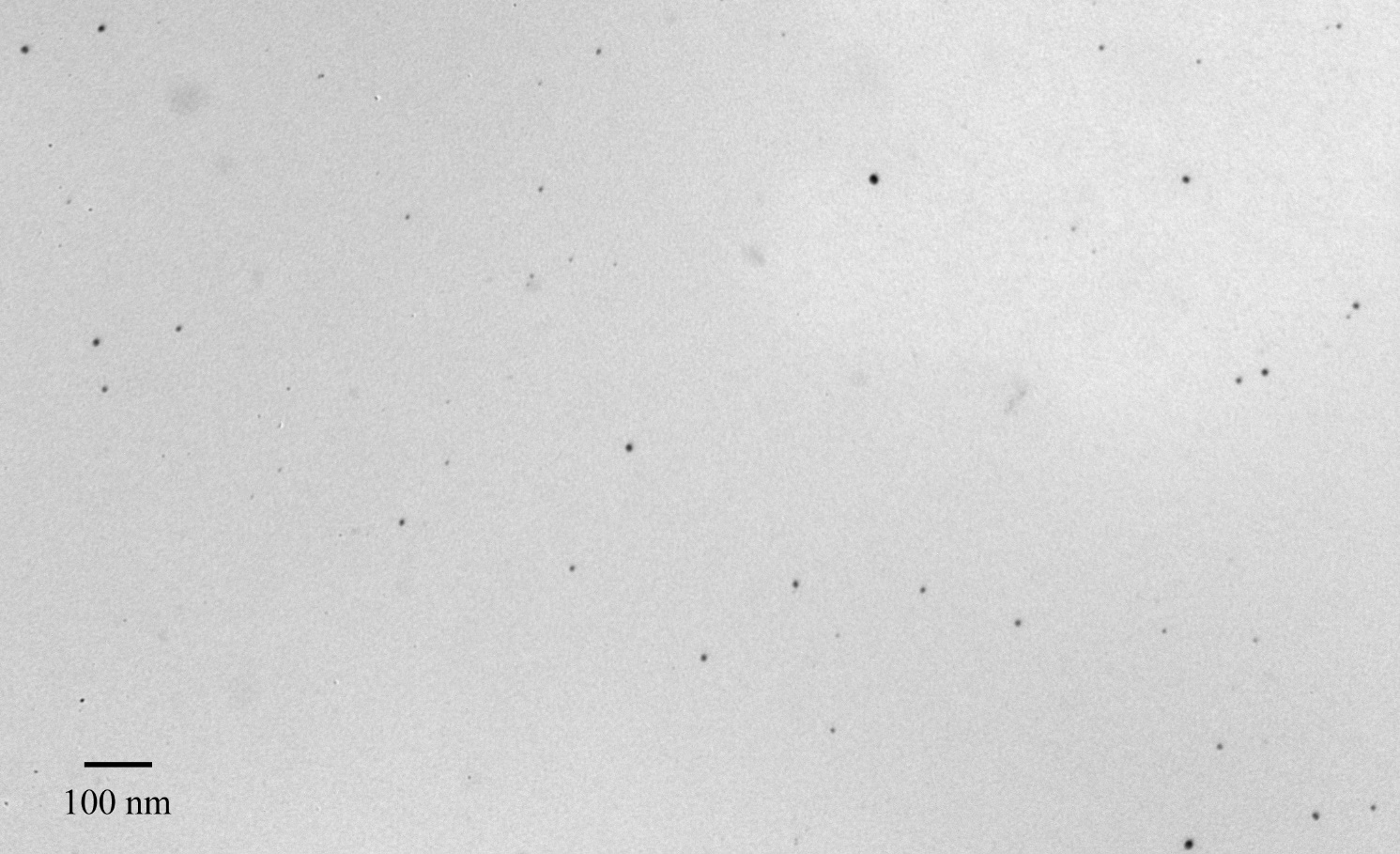


**Supplementary Figure S2**. Higher-resolution TEM image of AuNPs after 6 hours of biosynthesis.


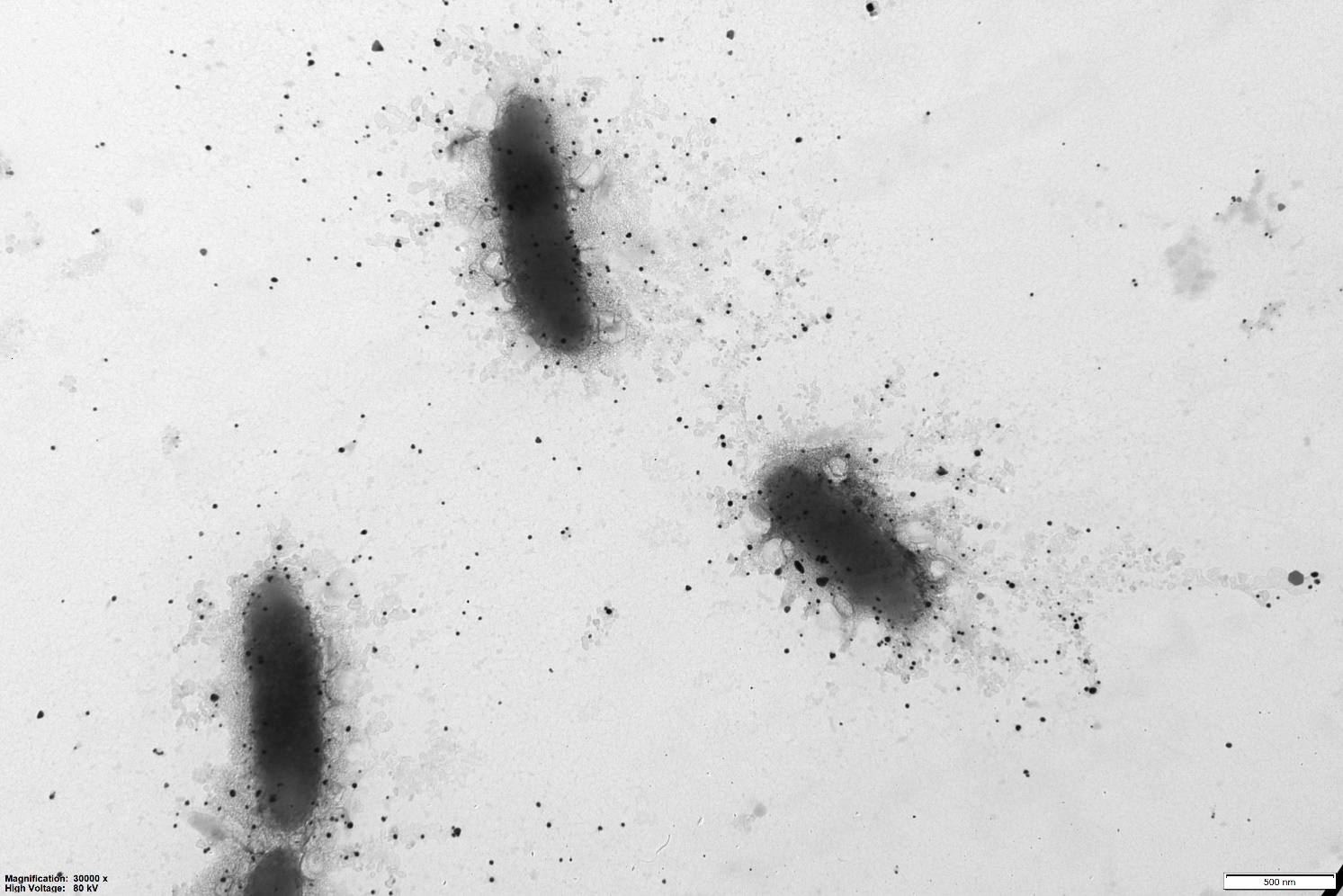


**Supplementary Figure S3**. Original TEM image of *Methylophilus* sp. F5P3.1m cells and AuNPs after 15 hours of biosynthesis with a larger field of view.


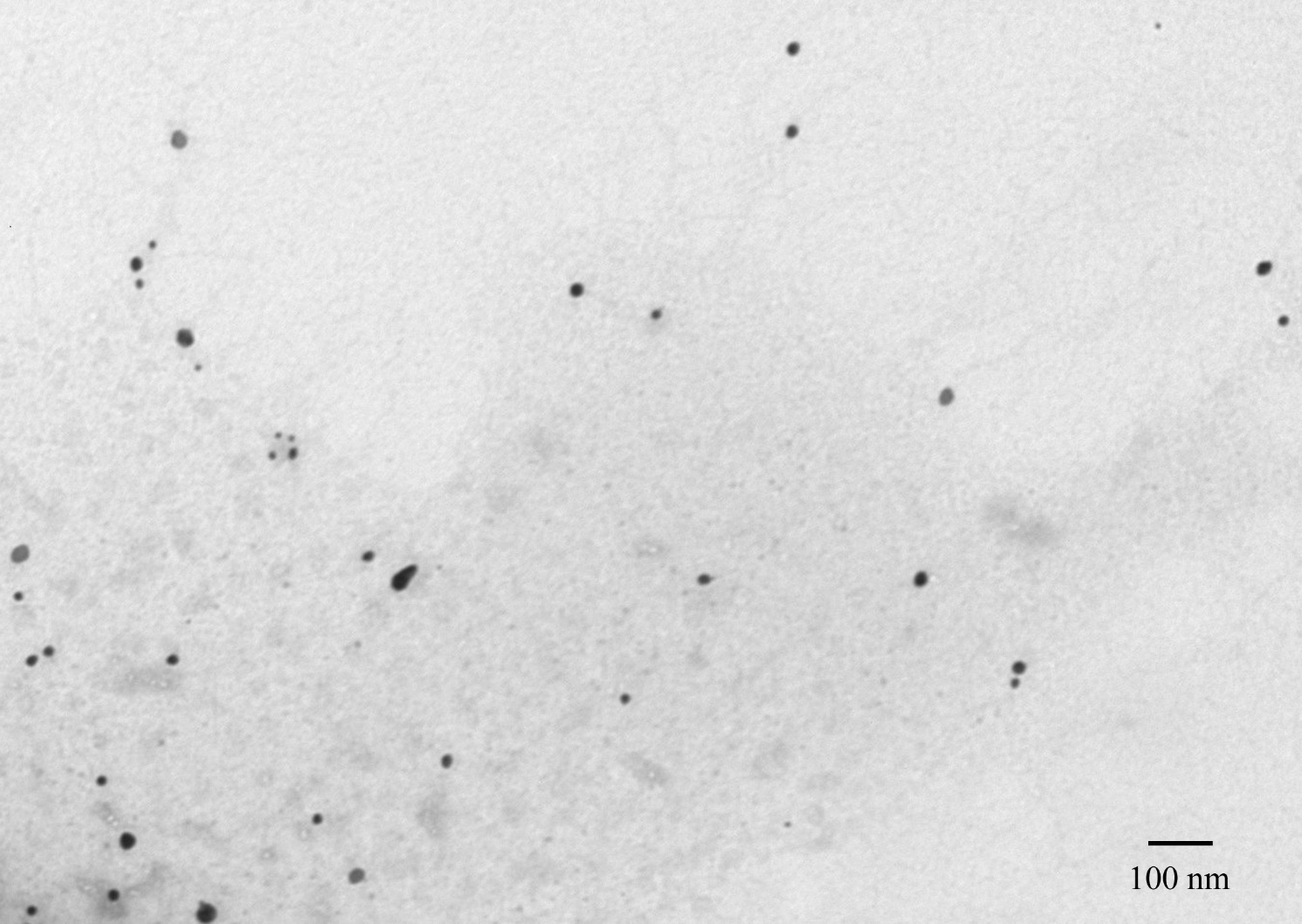


**Supplementary Figure S4**. Higher-resolution TEM image of AuNPs after 15 hours of biosynthesis.


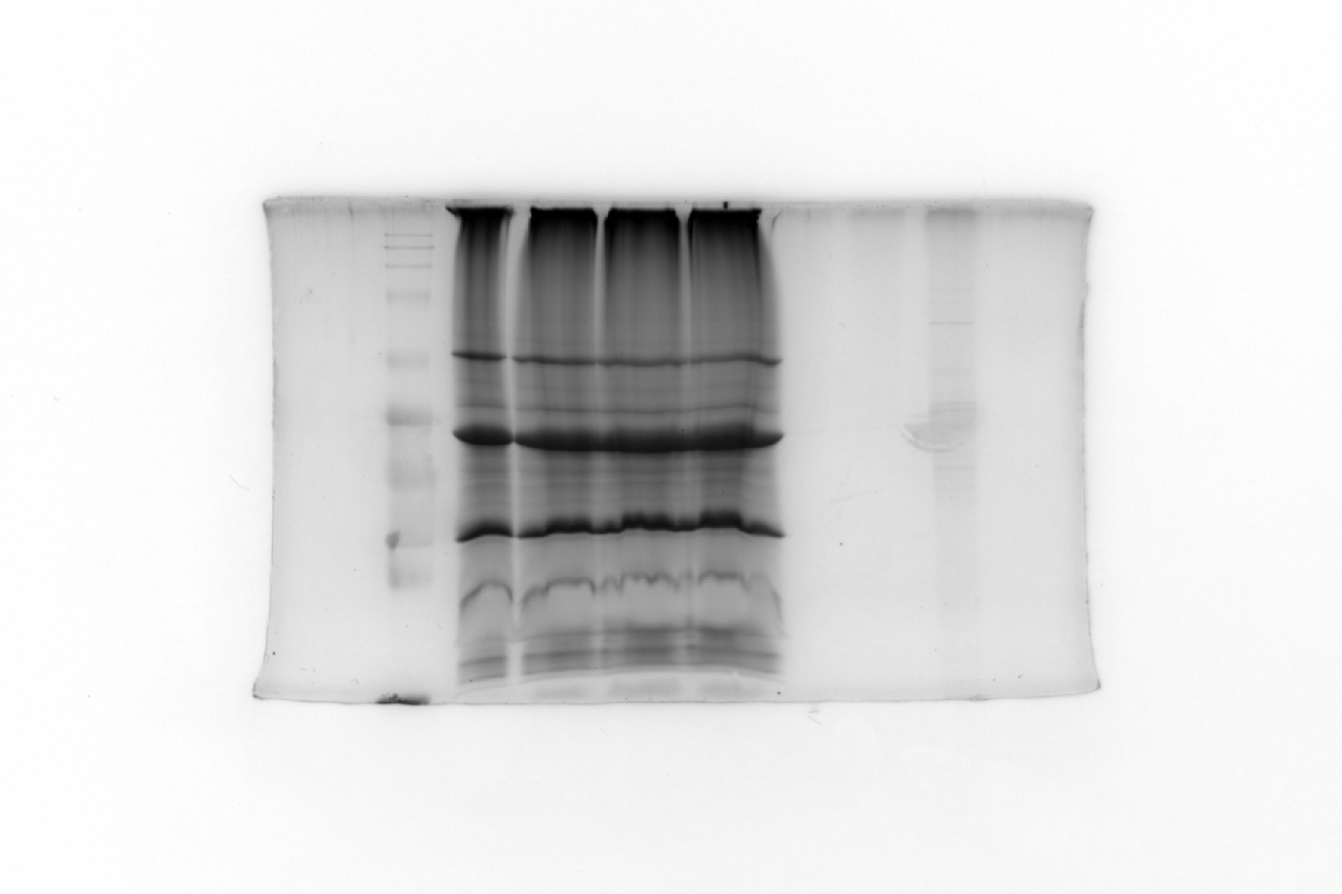


**Supplementary Figure 5.** Photo of protein electrophoresis for Figure 7 (original).
